# ROBUST LONGEVITY EFFECTS OF NOVEL COMPOUNDS TESTED BY THE CAENORHABDITIS INTERVENTION TESTING PROGRAM

**DOI:** 10.1093/geroni/igac059.2931

**Published:** 2022-12-20

**Authors:** Monica Driscoll, Brian Onken, Christine Sedore, Anna Coleman-Hulbert, David Hall, Gordon Lithgow, Patrick Phillips, Viviana Perez Montes

**Affiliations:** Rutgers, The State University of New Jersey, New Brunswick, New Jersey, United States; Rutgers University, Piscataway, New Jersey, United States; University of Oregon, Eugene, Oregon, United States; University of Oregon, Eugene, Oregon, United States; Buck Institute, Novato, California, United States; Buck Institute, Novato, California, United States; University of Oregon, Eugene, Oregon, United States; National Institute on Aging, Bethesda, Maryland, United States

## Abstract

Many efficacious interventions that promote mouse longevity (sirtuins, TOR, insulin signaling) have identification roots in invertebrate genetics. The Caenorhabditis Intervention Testing Program (CITP) was charged by the NIA to evaluate pharmacological interventions that promote healthy aging in a robust and reproducible manner across diverse genetic backgrounds of natural variant Caenorhabditis strains. The central premise of the CITP effort is that compounds that have strong effects across diverse genetic backgrounds have enhanced probability of translatability into pre-clinical research. Indeed, some CITP-verified compounds have been shown to promote healthspan and longevity in mouse models, in support of this fundamental premise. One example that supports the Geroscience hypothesis (anti-aging compounds will promote systemic health) is our finding that when HBX, the counterpart of one of the strongest C. elegans longevity interventions (ThioT), was tested in a comprehensive multi-laboratory collaboration (Buck Institute), a remarkable increase in bone health in aging mice emerged (PMID 33778327). Our current effort includes pursuing compound submissions from the larger scientific aging community, as well as identification of candidates via high-throughput screening of chemical libraries and data mining of peer-reviewed publications. We now evaluate whole-organism RNA sequence data to estimate the mode of action for successful interventions, and conduct mortality analysis from a generalized family of distributions on high-resolution automated lifespan data to determine whether a given intervention changes the rate or onset of aging. Our recent lifespan-altering discoveries include a small molecule, a proprietary compound, an isothiocyanate and a vitamin derivative. We will present on the breaking compound successes.

